# Case Report: Pulmonary benign metastasizing leiomyoma diagnosed through clinicopathological correlation

**DOI:** 10.3389/fmed.2026.1993661

**Published:** 2026-10-27

**Authors:** Shuai Zhao, Kai Wang, Wenkang Zong, Kunpeng Li, Zheng Wang, Han Zhang, Xin Li, Daqiang Sun

**Affiliations:** 1 Department of Thoracic Surgery, Tianjin Chest Hospital, Tianjin, China; 2 Department of Pathology, Tianjin Chest Hospital, Tianjin, China; 3 Tianjin Chest Hospital, Tianjin University, Tianjin, China

**Keywords:** clinicopathological correlation, differential diagnosis, pulmonary benign metastasizing leiomyoma, pulmonary nodules, uterine leiomyoma

## Abstract

**Background:**

Pulmonary benign metastasizing leiomyoma (PBML) is a rare condition characterized by histologically benign smooth-muscle tumors involving the lung, typically in women with a history of uterine leiomyoma. Its radiological features are nonspecific and may overlap with primary pulmonary neoplasms, metastatic malignancies, and other mesenchymal lesions. Diagnosis therefore depends on clinicopathological correlation integrating pulmonary pathological findings with the relevant gynecologic history.

**Case presentation:**

A 47-year-old woman with a history of uterine leiomyoma underwent evaluation for two right pulmonary nodules detected approximately 6 years and 8 months after uterine myomectomy. Follow-up CT showed stability of the right upper lobe nodule and slight enlargement of the right middle lobe nodule. Thoracoscopic wedge resections were performed for definitive diagnosis. Intraoperative frozen-section examination yielded different provisional interpretations for the two lesions and did not establish a definitive diagnosis. Permanent histopathological examination demonstrated spindle-cell tumors with smooth-muscle differentiation and hormone-receptor expression. Further clarification of the patient's remote gynecologic history, together with the pulmonary morphology and immunophenotype, supported the diagnosis of PBML. At the 1-month postoperative follow-up, no evidence of recurrence was identified, and annual CT surveillance was planned.

**Conclusion:**

PBML should be considered in the differential diagnosis of pulmonary nodules in women with a current or remote history of uterine leiomyoma. When pulmonary spindle-cell morphology is not diagnostic in isolation, integration of gynecologic history, imaging findings, permanent histopathology, and immunohistochemical findings is important for accurate diagnosis and avoidance of misclassification. This case highlights the value of clinicopathological communication when evaluating pulmonary mesenchymal lesions.

## Introduction

Pulmonary benign metastasizing leiomyoma (PBML) is a rare pulmonary manifestation of benign metastasizing leiomyoma (BML), characterized by histologically benign smooth-muscle lesions in the lung despite their metastatic distribution (1, 2). It occurs predominantly in women with a current or previous history of uterine leiomyoma (3). Pulmonary lesions are often incidentally detected as solitary or multiple well-circumscribed nodules, and affected patients may remain asymptomatic for prolonged periods (4, 5). Because these radiological findings are nonspecific, PBML may overlap with primary pulmonary neoplasms, metastatic malignancies, and other benign or low-grade mesenchymal lesions (2, 4). Recognition therefore requires correlation of pulmonary pathological findings with the patient's gynecologic history and clinical context (2).

The diagnosis of PBML can remain challenging in routine practice because pulmonary findings are nonspecific and the relevant gynecologic history may be remote or not initially appreciated during evaluation of lung lesions (2, 4). Histopathological and immunohistochemical findings can establish smooth-muscle differentiation and hormone-receptor expression, but their interpretation requires appropriate clinical context (3, 5). Effective communication between clinicians and pathologists is therefore important when evaluating pulmonary mesenchymal lesions in women with a history of uterine leiomyoma.

Here, we report a case of PBML presenting as two pulmonary nodules with different radiological behavior in a woman with a remote history of uterine leiomyoma. The case illustrates the diagnostic process from thoracic surgical evaluation and intraoperative assessment to permanent pathological examination and clinicopathological correlation. By highlighting how a remote gynecologic history became relevant to the interpretation of pulmonary pathological findings, this report emphasizes the importance of integrating clinical history, imaging, and pathology when evaluating pulmonary mesenchymal lesions.

## Case description

A 47-year-old woman was referred after pulmonary nodules were detected during a routine health examination. She had no respiratory symptoms, including chest discomfort, cough, or sputum production. Her medical history included uterine myomectomy performed in November 2018. According to the available pathology report, the uterine lesions were diagnosed as multiple leiomyomas, with negative p16 and p53 expression and a low Ki-67 index of approximately 2%. The original uterine histological slides and tissue blocks were unavailable for review, precluding histological re-evaluation and direct morphological comparison with the pulmonary lesions.

Chest computed tomography (CT) performed in July 2025 revealed well-circumscribed solid nodules with smooth margins in the right upper lobe (RUL) and right middle lobe (RML), without obvious spiculation or cavitation. Follow-up CT approximately four months later showed slight enlargement of the RML lesion, whereas the RUL lesion showed no obvious interval change (Figure 1). The persistence of both nodules, with interval enlargement of the RML lesion, prompted surgical resection for definitive diagnosis.

**Figure 1:**
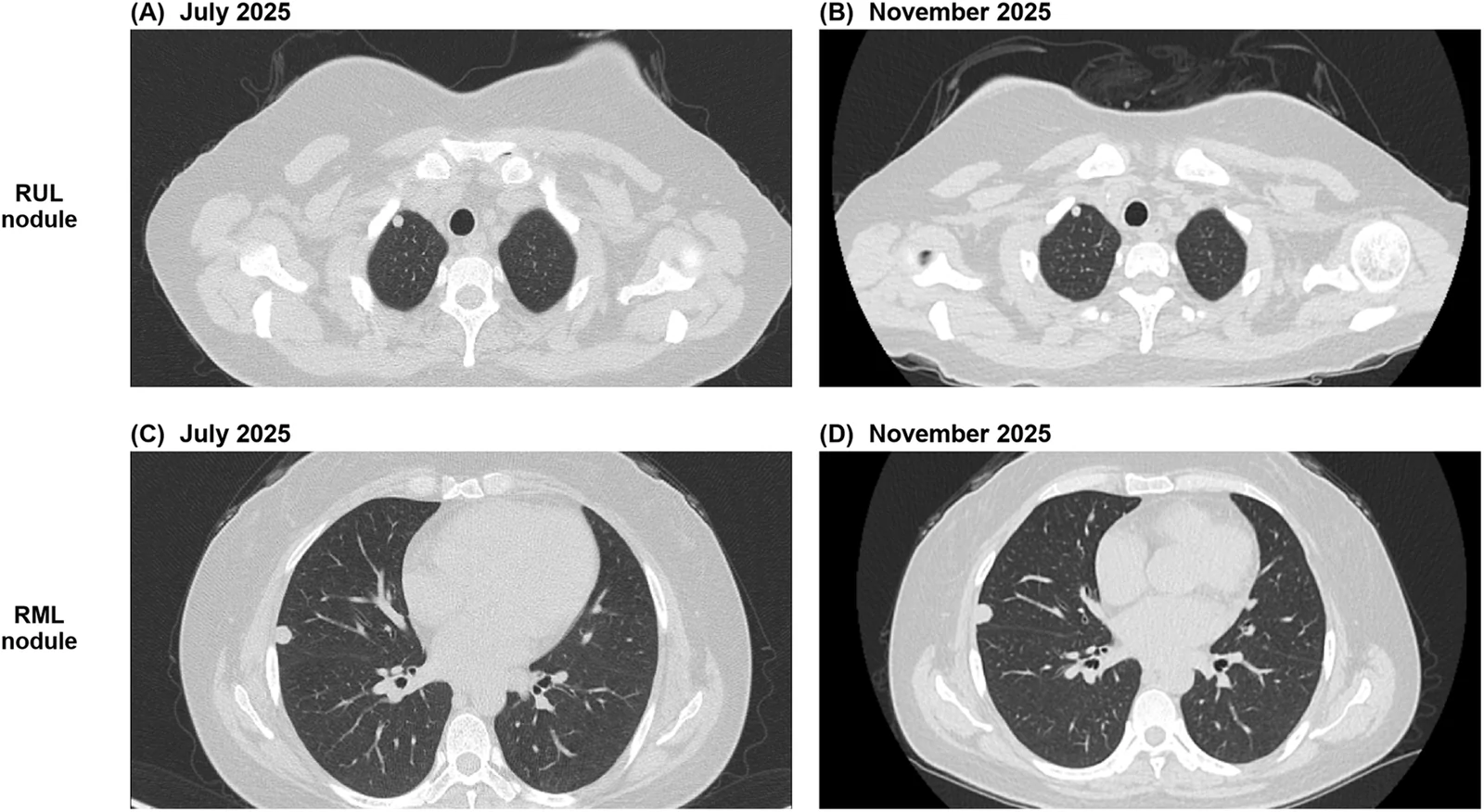
Radiological evolution of two well-circumscribed solid pulmonary nodules. **(A,B)** Chest CT images of the right upper lobe (RUL) nodule obtained in July 2025 and November 2025, respectively, showing a well-circumscribed solid nodule with smooth margins and no obvious interval change. **(C,D)** Chest CT images of the right middle lobe (RML) nodule obtained in July 2025 and November 2025, respectively, showing a well-circumscribed solid nodule with smooth margins and slight enlargement during follow-up. Neither lesion showed obvious spiculation or cavitation.

### Diagnostic assessment and surgical intervention

Image-guided biopsy was considered; however, the small size of both lesions, particularly the 0.5-cm RUL nodule, raised concerns about obtaining sufficient tissue with preserved architecture for comprehensive pathological and immunohistochemical evaluation. Given the patient's suitability for surgery and the peripheral location of both nodules, video-assisted thoracoscopic wedge resection was performed to obtain adequate tissue for definitive diagnosis. Thoracoscopic examination revealed a subpleural RML nodule with a smooth surface, no pleural indentation, firm consistency, and slight lobulation (Figure 2A). The gross appearance of the resected RML nodule is shown in Figure 2B. Intraoperative frozen-section examination did not establish a definitive diagnosis: the RML nodule was considered suggestive of a mesenchymal hamartoma, whereas the RUL nodule was interpreted as a likely benign small round-cell tumor, although metastatic disease could not be excluded.

**Figure 2:**
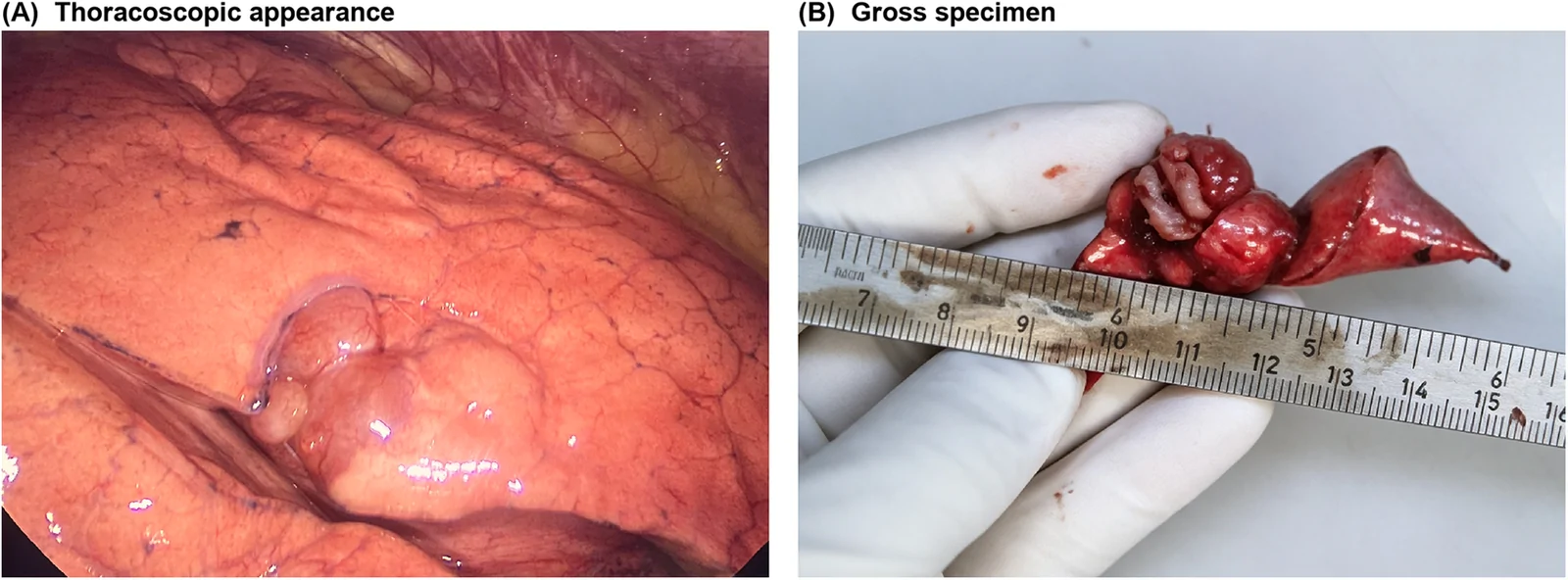
Thoracoscopic findings and gross specimen of the right middle lobe pulmonary nodule. **(A)** Intraoperative thoracoscopic view showing a subpleural nodule in the right middle lobe (RML) with a smooth surface and slight lobulation. **(B)** Gross appearance of the resected RML pulmonary nodule.

    Permanent examination showed spindle-cell tumors measuring 1.5 × 1.2 cm in the RML and 0.5 × 0.4 cm in the RUL, both arranged in intersecting fascicles (RML shown in Figures 3A,B). The RML lesion was positive for vimentin, desmin, SMA, ER, and PR, with a Ki-67 index below 5%. The RUL lesion showed scattered SMA positivity, was negative for desmin, and was positive for ER and PR, with a Ki-67 index below 10%. Both lesions were negative for broad-spectrum cytokeratin, TTF-1, and p40. Overall, the morphological and immunophenotypic findings supported smooth-muscle differentiation (Figures 3C–G).

**Figure 3:**
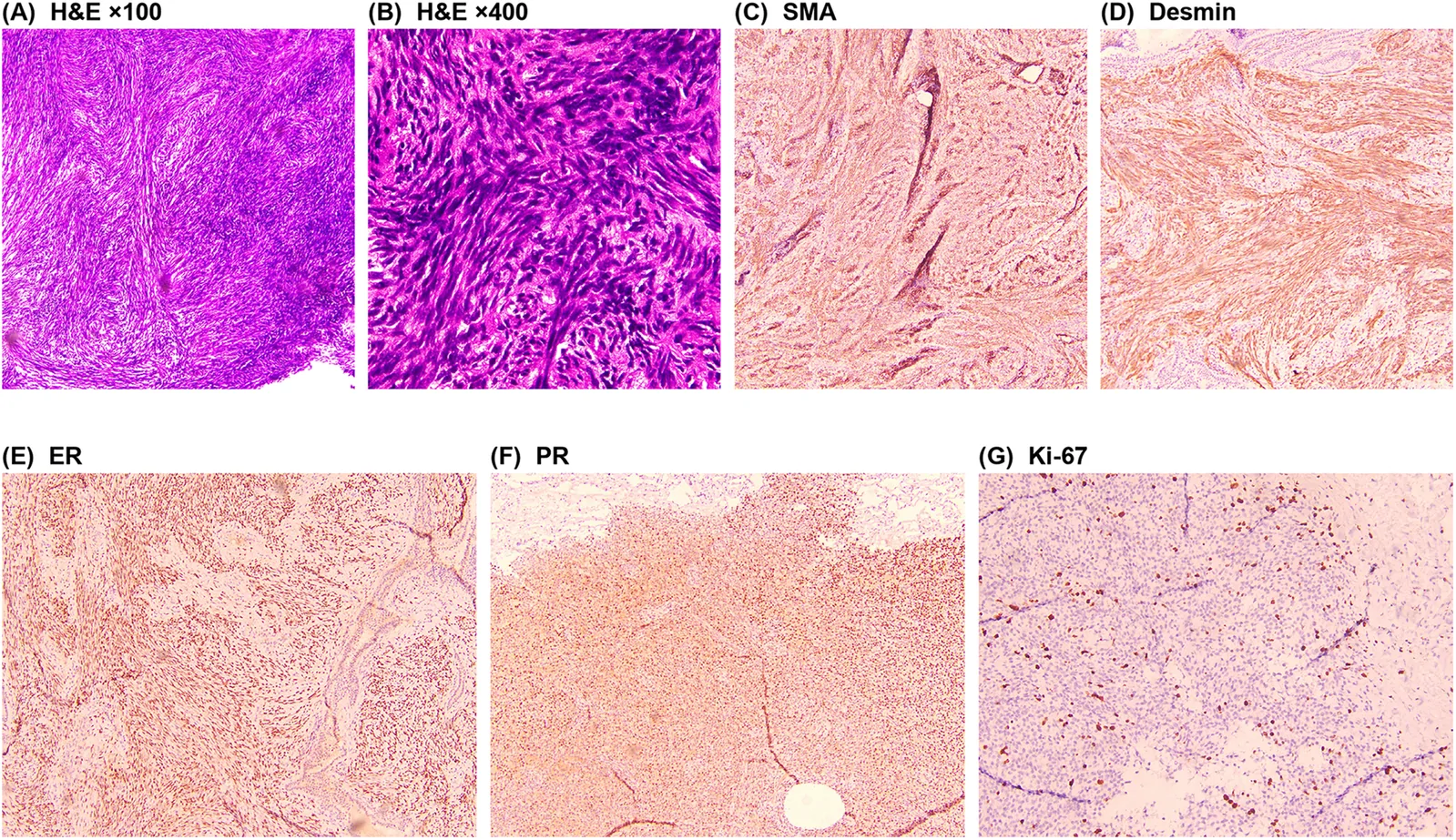
Histological and immunohistochemical findings of the right middle lobe pulmonary nodule. All panels were obtained from the RML lesion. **(A,B)** Hematoxylin and eosin staining showing spindle-cell tumor arranged in intersecting fascicles. **(C)** Immunohistochemical staining for SMA. **(D)** Immunohistochemical staining for Desmin. **(E)** Immunohistochemical staining for ER. **(F)** Immunohistochemical staining for PR. **(G)** Ki-67 staining showing low proliferative activity.

During permanent-section evaluation, the pathology team requested further clarification of the patient's gynecologic history. Review of the remote uterine myomectomy and the available documentation of benign leiomyoma provided essential clinicopathological context. Integration of the historical uterine pathology information, pulmonary spindle-cell morphology, hormone-receptor expression, and smooth-muscle immunophenotype supported the diagnosis of PBML. Because the original uterine slides were unavailable for review, the correlation was based on historical and clinicopathological information rather than direct paired histological comparison. The resection margins were negative for tumor involvement.

### Follow-up and outcomes

The postoperative course was uneventful. Postoperative management options were discussed with the patient. Because both pulmonary nodules had been completely resected with negative margins and the patient was asymptomatic, surveillance was selected rather than immediate additional treatment. No adjuvant therapy was initiated. At the 1-month follow-up, there was no evidence of recurrence, and annual chest CT surveillance was planned (Figure 4).

**Figure 4:**
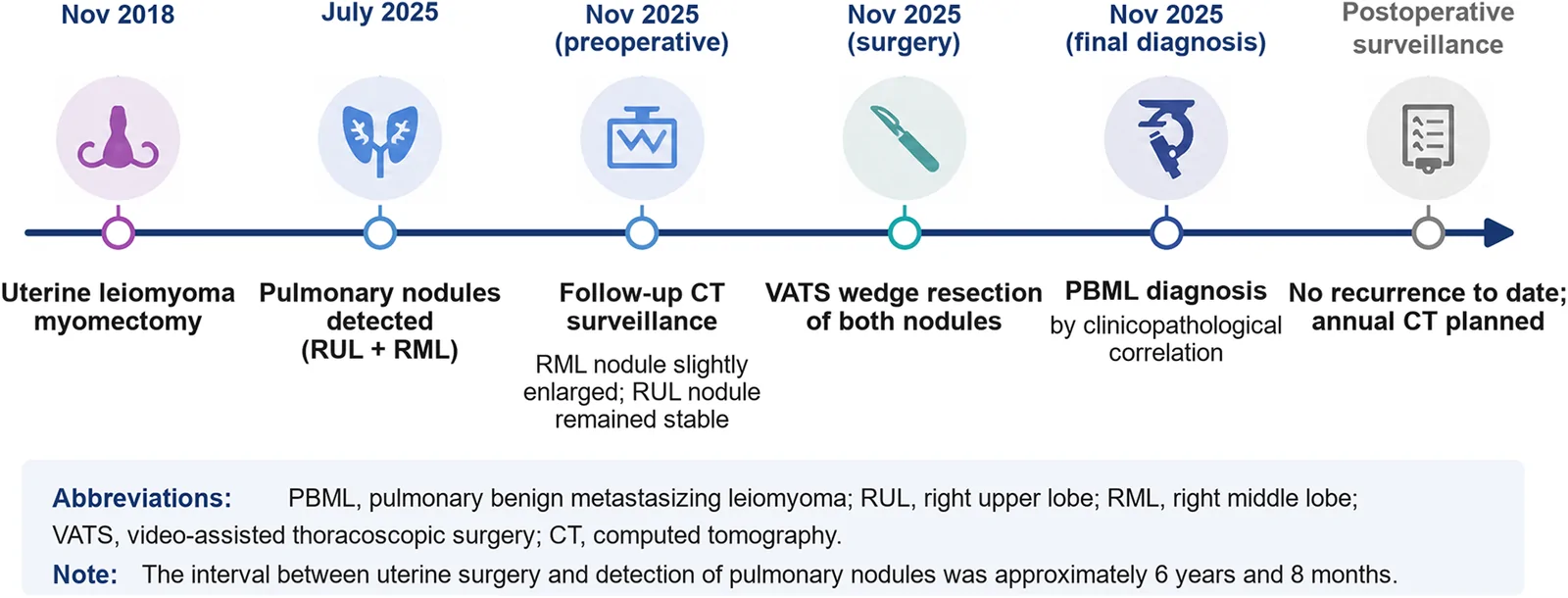
Clinical timeline of uterine and pulmonary disease. Timeline illustrating uterine myomectomy in November 2018, detection of pulmonary nodules in July 2025, radiological follow-up showing interval changes in the pulmonary nodules, subsequent video-assisted thoracoscopic surgical biopsy leading to the diagnosis of PBML, and postoperative surveillance with planned annual CT follow-up.

## Discussion

PBML is rare and may be overlooked in thoracic practice because its radiological presentation overlaps with more common pulmonary conditions (2, 3). Although multiple bilateral solid nodules are frequently described, solitary lesions and other atypical pulmonary imaging patterns have also been reported (2, 6). In the present case, the two right-sided solid nodules were well circumscribed, with smooth margins and no obvious spiculation or cavitation; the RUL lesion showed no obvious interval change, whereas the RML lesion enlarged slightly. This nonspecific appearance raised a differential diagnosis of primary pulmonary neoplasm, pulmonary metastases, granulomatous or other inflammatory nodules, hamartoma, and other benign mesenchymal tumors. Neither CT morphology nor short-term changes were specific for PBML. Intraoperative frozen-section examination likewise yielded different provisional interpretations for the two lesions and did not establish a definitive diagnosis. These findings illustrate why radiological appearance and intraoperative morphology alone may be insufficient to establish the diagnosis of PBML and why definitive interpretation may require permanent histology, immunophenotypic findings, and relevant clinical history.

Primary pulmonary leiomyoma was considered because of its overlapping smooth-muscle morphology and immunophenotype. The history of uterine leiomyoma, two pulmonary smooth-muscle lesions, and ER/PR positivity in both favored PBML in the overall clinicopathological context but did not establish uterine origin. The original uterine material was unavailable for histological re-evaluation and direct morphological comparison, and paired molecular analysis was not performed; uterine origin therefore remained unconfirmed. The pulmonary lesions also differed in their smooth-muscle marker profiles: the RML lesion was positive for vimentin, desmin, and SMA, whereas the RUL lesion showed only scattered SMA positivity and was negative for desmin. Both nevertheless showed spindle-cell morphology with an intersecting fascicular growth pattern. Interpretation of the RUL lesion relied on its morphology, immunophenotype, and clinical context despite the differing smooth-muscle marker profiles (3, 5). The shared morphology and hormone-receptor expression supported this clinicopathological interpretation but did not establish a common clonal origin of the two pulmonary lesions. During permanent-section evaluation, further clarification of the patient's remote gynecologic history substantially refined the differential diagnosis. The differing intraoperative and permanent-section interpretations should be understood in the context of limited sampling and incomplete immunohistochemical and clinical information during frozen-section evaluation, rather than as contradictory findings. This sequence underscores the importance of communication between clinicians and pathologists: in women with unexplained pulmonary spindle-cell tumors or solid pulmonary nodules of uncertain origin, careful review of prior gynecologic history and available pathology records may provide essential diagnostic context.

The interval between uterine and pulmonary disease is a clinically relevant feature of BML (3). In the present patient, pulmonary nodules were detected approximately 6 years and 8 months after uterine myomectomy, representing a frequently reported sequence in which uterine disease precedes pulmonary manifestation. Prolonged intervals between uterine surgery and pulmonary manifestation have been repeatedly described and should not be considered exceptional (3, 7). However, the timing of radiological detection should not be equated with the timing of biological dissemination. Reported temporal relationships are heterogeneous, including pulmonary disease detected after uterine surgery, around the time of uterine disease, or in patients without prior gynecologic surgery (3, 5). Prior uterine surgery is therefore not a prerequisite for pulmonary presentation. These temporal relationships are clinical observations rather than direct evidence of mechanism. In our patient, the prolonged interval is compatible with possibilities such as dormant dissemination or slow hormone-responsive growth, but delayed detection of a pre-existing pulmonary lesion cannot be excluded. Chronology alone therefore cannot establish the timing or mechanism of dissemination.

The pathogenesis of BML remains unresolved (2, 3). Proposed explanations include hematogenous dissemination of benign-appearing uterine smooth-muscle cells, iatrogenic dissemination, hormonally driven growth at distant sites, and misclassification of an indolent low-grade malignant smooth-muscle neoplasm (2, 3). The long and highly variable latency of PBML, together with reports in women without prior uterine surgery, argues against a single surgery-dependent explanation (3, 5). Molecular studies have provided evidence that at least a subset of BML represents clonal dissemination from a uterine smooth-muscle tumor (8, 9). However, these findings do not establish a uniform mechanism of dissemination, dormancy, or delayed growth across all patients. In this case, the original uterine pathological material was unavailable, and neither uterus–lung nor lung–lung paired molecular testing was performed. Thus, neither uterine origin nor a common clonal origin of the pulmonary lesions could be directly demonstrated; the diagnosis remained based on clinicopathological correlation.

There is no universally accepted postoperative adjuvant treatment for PBML, and management should be individualized according to disease extent, symptoms, progression, and hormone-receptor status. Reported approaches include observation, surgical resection, ovarian suppression or ablation, and endocrine therapy (3, 10). ER and PR expression, together with documented responses to hormonal manipulation, supports hormone sensitivity in at least a subset of PBML (3, 10); however, receptor positivity alone does not mandate immediate systemic treatment. Hormone-directed therapy may be considered for progressive, unresectable, multifocal, or symptomatic disease, whereas surveillance may be appropriate for limited, indolent, asymptomatic disease. Complete resection with negative margins and the absence of symptoms supported surveillance rather than immediate additional therapy. After discussion with the patient, annual chest CT was planned. Follow-up was limited to 1 month, precluding assessment of long-term disease control. Continued CT surveillance is appropriate because PBML may follow a prolonged clinical course and delayed progression has been reported (7, 10).

This report has several limitations. First, as a single case report, the findings cannot be generalized to all patients with PBML. Second, the original uterine pathology slides and tissue blocks were unavailable, precluding histological re-evaluation of the uterine lesions and direct morphological comparison with the pulmonary tumors. In addition, paired molecular analysis was not performed between the uterine and pulmonary lesions or between the two pulmonary lesions; therefore, their clonal relationships could not be directly demonstrated. Third, postoperative follow-up remains limited, and longer observation is required to evaluate long-term clinical outcomes. These limitations should be considered when interpreting the clinical and biological implications of this case.

## Patient perspective

The patient was concerned about the nature of the pulmonary nodules when they were detected during routine examination despite the absence of respiratory symptoms. She considered obtaining a definitive diagnosis important and agreed to thoracoscopic surgical biopsy. After being informed of the diagnosis and the planned surveillance strategy, she expressed relief and understood the need for continued follow-up with annual chest CT.

## Conclusion

PBML should be considered in the differential diagnosis of pulmonary nodules in women with a current or remote history of uterine leiomyoma. Accurate diagnosis often requires integration of clinical history, imaging findings, permanent histopathology, and immunohistochemical features, particularly when pulmonary morphology is not diagnostic in isolation. Effective communication between clinicians and pathologists may help avoid misclassification of pulmonary mesenchymal lesions. Long-term surveillance remains appropriate given the typically indolent but potentially prolonged and variable clinical course of PBML.

## Data Availability

The raw data supporting the conclusions of this article will be made available by the authors, without undue reservation.
